# Study on the multidrug resistance and transmission factors of *Staphylococcus aureus* at the ‘animal–environment–human’ interface in the broiler feeding cycle

**DOI:** 10.3389/fmicb.2025.1495676

**Published:** 2025-02-12

**Authors:** Fangyuan Hu, Yaopeng Liu, Lin Wang, Juan Wang, Na Liu, Yan Li, Xiaoxiao Duan, Junwei Wang, Mingzhe Lu, Junhui Liu, Zhina Qu, Keguang Han

**Affiliations:** ^1^College of Veterinary Medicine, Shanxi Agricultural University, Jinzhong, China; ^2^China Animal Health and Epidemiology Center, Qingdao, China; ^3^Qingdao Animal Disease Prevention and Control Center, Qingdao, China

**Keywords:** *Staphylococcus aureus*, broiler, AMR, MDR, one health

## Abstract

Multidrug-resistant *Staphylococcus aureus* (*S. aureus*) poses an increasingly serious threat to agricultural safety and public health. Based on the concept of “One Health,” this study analyzed the multidrug resistance and transmission factors of *S. aureus* isolated from the “animal–environment–human” interface during one feeding cycle of commercial broilers in China by using antimicrobial susceptibility testing and whole genome sequencing (WGS) technologies. The results showed that in stage 1, the isolation rate of *S. aureus* was 1.32% (6/453), that of workers was 25.0% (4/16), and that of environmental samples was 0.69% (2/287), and the multidrug resistance rate was 83.33%. After one feeding cycle, the isolation rate of *S. aureus* (221/772, 28.63%) increased significantly (*p* < 0.01) during stage 2, and the multidrug resistance rate was as high as 97%. The resistance rates to eight drugs including erythromycin, clindamycin, enrofloxacin, ofloxacin, doxycycline, florfenicol, tylosin, and tilmicosin were elevated, but the differences were not significant (*p* > 0.05). ST398 (79.13%) was the dominant strain in both stages, which was prevalent in 11 types of samples from 3 sources and clustered in the same sub-branch of the single-nucleotide polymorphism (SNP) evolutionary tree. The loci difference between the strains ranged from 1 to 541, with SNPs of less than 10 between the human strains of stage 1 and the three sources in stage 2. The 42 representative strains carried mobile elements, mainly plasmid replicons (10 types), transposons (3 types), and 20 antibiotic resistance genes in 9 classes. A total of 10 ST398 strains exhibited the *fosB* gene for fosfomycin resistance, and 6 ST9 strains from stage 2 exhibited the *mecA* resistance gene. The SNP evolutionary analysis revealed that the *fosB* resistance gene might have been brought in by workers during stage 1. This study revealed the critical impact of environmental residual and worker-carried *S. aureus*, as well as the transmission of antibiotic resistance in stage 1. It highlighted the importance of the “One Health” approach and biosecurity measures and provided recommendations for the prevention of the spread of pathogens and resistance.

## 1 Introduction

*Staphylococcus aureus* (*S. aureus*) is a common zoonotic pathogen, which is not only widely distributed in the environment but also commonly exists in the skin, hair, nasal cavity, and throat of warm-blooded animals (Zhou et al., 2017; Algammal et al., 2020; Liu et al., 2021). It is the main cause of human and animal infections, leading to diseases such as mastitis, endocarditis, septicemia, and toxic shock syndrome (Wang and Muir, 2016). Its prevalence rate in humans, pigs, and broilers can be as high as 20–30%, 42, and 90% (Haag et al., 2019), respectively, positioning it as one of the leading causes of bacterial foodborne diseases (Bowman et al., 2016). In addition, it is listed as a high-priority pathogen in the WHO Bacterial Priority Pathogens List (WHO Bacterial Priority Pathogens List, 2024). With the widespread and irrational use of antimicrobial agents, the drug resistance of *S. aureus* has become increasingly serious (Subramanya et al., 2021), especially with the emergence of methicillin-resistant *Staphylococcus aureus* (MRSA), which poses a major challenge to public health. The Centers for Disease Control and Prevention (CDC) estimates that the consumption of *S. aureus*-contaminated animal-related foods causes approximately 241,000 illnesses in the United States each year (Zeaki et al., 2019), with approximately 80,000 MRSA cases occurring annually, resulting in approximately 11,000 deaths (Kochanek et al., 2016). Research conducted in Canada revealed that all chicken-derived strains exhibited resistance to three or more antibiotics (Bernier-Lachance et al., 2020). Studies in the United States indicated that 78.4% of strains isolated from poultry samples were multidrug-resistant (Waters et al., 2011). China reported multidrug-resistant rates as high as 71.9% among broilers from the Chongqing region (Dong et al., 2023). In recent years, with the emergence and application of the “One Health” concept, many studies have found a closer association between *S. aureus* infections in animals and humans (Hanning et al., 2012; Crespo-Piazuelo and Lawlor, 2021). A study in the Netherlands revealed that 35% of *S. aureus* strains were found in farm broilers and 20% in slaughter workers (Mulders et al., 2010). Additionally, environmental factors have also been widely concerned (McCallum et al., 2010; Bengtsson-Palme et al., 2018). A Taiwan Province study showed that the multidrug resistance rate of *S. aureus* in the farm environment reached as high as 100% (Tao et al., 2021).

China is the largest producer and consumer of chicken in the world (Waters et al., 2011; Wang et al., 2023). Factors that affect the spread and antimicrobial resistance (AMR) of bacteria are complex and diverse. There is an urgent need to reduce the contamination of *S. aureus* and its multidrug-resistant bacteria (MDRB) and the spread of “superbugs (MRSA)” in animal products. Strengthening source control is a crucial measure to ensure the hygiene and safety of animal-derived food. The concept of “One Health” has become a consensus for addressing the challenge of AMR by promoting the common health of “animal, environment, and human.” This study employs the “One Health” concept to conduct a comprehensive analysis of the factors influencing the transmission of *S. aureus* and its drug-resistant characteristics among farming workers during the broiler feeding cycle. The findings are intended to serve as a reference for the prevention and control of multidrug-resistant *S. aureus* (MDRSA) in food sources.

## 2 Article types

Antimicrobials, Resistance, and Chemotherapy.

## 3 Materials and methods

### 3.1 Strain collection and identification

From September 2022 to May 2023, a total of 1,225 samples were collected from 3 commercial broiler farms in China, including chicks and broilers (throat swabs), environmental samples (air, drinking water, troughs/cages, floors, flies, transportation tools, feed, feces, and soils), and farm workers (throat swabs and hand swabs). The sample collection process was divided into two stages (stage 1, day 1; stage 2, day 41). In stage 1, samples from the environment and farm workers were collected 1 day before the chicks entered the coop. For the convenience of analysis, the samples of chicks (first day arrived in coop) were also included in stage 1. In stage 2, the samples were collected 2 days before the broilers (day 41) left. Environmental samples were collected using sterile cotton swabs soaked in sterile saline. The swab samples were stored in Cary–Blair Transport Medium. All samples were transported and stored at 4°C.

The collected samples were placed in 15 mL centrifuge tubes containing 7 mL of 7.5% NaCl broth and incubated at 37°C for 18 h. Subsequently, the samples were inoculated onto *S. aureus* chromogenic medium for preliminary screening. Single colonies were then transferred to trypticase soy agar (TSA) medium and incubated at 37°C for 18 to 20 h. Then, single colonies were picked and identified using an automated mass spectrometry microbial identification system MS1000. The quality control strain (ATCC 29213) was provided by the Pathogenic Microbial Surveillance Unit of the China Animal Health and Epidemiology Center.

### 3.2 Antimicrobial sensitivity test

Minimum inhibitory concentrations (MICs) of 19 kinds of antimicrobial drugs in 13 classes were determined using the micro broth dilution method, and 139 representative strains from different sources and sample types were selected, considering the drug resistance breakpoint by Clinical Laboratory Standards Institute (CLSI) (2023) as a reference. The antibiotics included penicillin (PEN), amoxicillin/clavulanic acid (AMC), erythromycin (ERY), clindamycin (CLI), enrofloxacin (ENR), ofloxacin (OFL), ceftiofur (CEF), cefoxitin (CFX), sulfisoxazole (SIS), oxacillin (OXA), vancomycin (VAN), cotrimoxazole (SXT), doxycycline (DOX), florfenicol (FFC), tamoxifen (TIA), tilmicosin (TIL), gentamicin (GEN), linezolid (LZD), and tetracycline (TET). ATCC 29213 and normal saline were treated as the positive control and negative control, respectively. The positive control had bacterial growth, and the negative control had aseptic growth, which was effective in explaining the results. Multidrug resistance (MDR) was defined as resistance to three or more classes of antibiotics. Furthermore, methicillin resistance was confirmed by polymerase chain reaction (PCR) amplification of the *mecA* gene (Ullah et al., 2021).

### 3.3 Multilocus sequence typing (MLST) and analysis

Seven pairs of housekeeping genes (*arcC*, *aroE*, *glpF*, *gmk*, *pta*, *tpi*, and *yqiL*) of *S. aureus* were detected using PCR (Enright et al., 2000), and PCR amplification products were sent to Tsingke Biotechnology Co. (Beijing, China). MegAlign and EdiSeq software were utilized for sequence reading. The sequencing results were compared and sheared, the allelic profiles and STs of each isolate were assigned based on the MLST database,^1^ and the MLST minimum spanning tree was constructed using BioNumerics 7.6 software.

### 3.4 Whole genome sequencing

According to the results of MLST, 42 representative strains of different types (6 from broilers, 23 from the environment, and 13 from humans) were selected, and each strain was sequenced by MGI DNBSEQ-T7 and MGISEQ-2000RS at Sangon Biotech (Beijing, China). (1) Genomic DNA extraction: DNA was extracted using a MagPure Bacterial DNA Kit (D6361-02, Magen, China); (2) library construction and sequencing was performed using MGI DNBSEQ-T7, and the whole genome DNA was randomly fragmented to an average size of 200–400 bp. The selected fragments underwent end-repair, 3′ adenylation, adapters-ligation, and PCR amplification. After purification with the magnetic beads, the library was qualified using a Qubit 4.0 fluorometer, and the length of the library was assessed via the 2% agarose gel electrophoresis. The qualified libraries were sequenced on MGI DNBSEQ-T7. After sequencing, raw reads were filtered via Trimmomatic (v0.36) by removing adaptors and low-quality reads, and then clean reads were obtained. Genome assembly was performed using SPAdes(v3.15), and Gapfiller (v1.11) was used to fill gaps. The whole gene-sequencing results of all strains were submitted to the GenBank database, and the BioProject ID (PRJNA1184813) was obtained. Gene annotation was performed using bakta (v1.10.3), and gene backgrounds were mapped based on the annotation results viewed by Snapgene.

### 3.5 Statistical analysis

Pearson correlation analysis was performed using the SPSS 26.0 statistics software (*p* > 0.05 was labeled ns, 0.01 < *p* < 0.05 was labeled *, and *p* < 0.01 was labeled **). Resistance gene comparison was performed using the ResFinder database,^2^ with a minimum gene coverage of 80% and a minimum identity cutoff of 90%. Spa typing was performed using the database website spaTyper.^3^ Mobile genetic elements were re-screened using MobileElementFinder v1.0.3. The core genome evolutionary tree was constructed using BioNumerics v7.6 software (Applied Maths, Sint-Martens-Latem, Belgium) for the 42 strains selected in this study, and the constructed core genome evolutionary tree was embellished and visualized in the ChiPlot online tool.^4^ Single-nucleotide polymorphism (SNP) analysis of the 42 strains was performed through the BacWGSTdb database,^5^ with BA02176_CP003808_ST398 and BA01611_CP019945_ST9 as the reference strains. A heat map was produced using ChiPlot Phylogenetic Tree 2.6.1.^6^

## 4 Results

### 4.1 Prevalence of *S. aureus* in different types of samples

During one feeding cycle of broilers, 227 strains of *S. aureus* were isolated from 1,225 samples obtained from the animal, environment, and human sources (Table 1). The isolation rate of *S. aureus* was 1.32% (6 out of 453) in stage 1. No strains were isolated from the animal samples (chicks) (0 out of 150). The isolation rate of the environmental samples was 0.69% (2 out of 287), of which two strains were from soil (1 out of 15, 6.67%) and trough/cage samples (1 out of 120, 0.83%). However, the isolation rate from the human samples was 25% (4 out of 16), including throat and hand swabs (2 out of 8). In stage 2, the isolation rate of *S. aureus* increased to 28.63% (221 out of 772), which was extremely significantly higher than that in stage 1 (*p* < 0.01). The isolation rate of broiler samples was 30.74% (83 out of 270), which was extremely significantly higher than that in stage 1 (*p* < 0.01). The isolation rate of environmental samples was 25.21% (117 out of 464), including cage/trough samples (42.35%, 72 out of 170), floor samples (33.33%, 20 out of 60), and air samples (25%, 10 out of 40), which were extremely significantly higher than those in stage 1 (*p* < 0.01). Although the isolation rates from transport, soil, feed, and feces samples were higher than those in stage 1, the differences were not statistically significant (*p* > 0.05). Furthermore, the isolation rate among farm workers was 55.27% (21 out of 38), which was significantly higher (*p* < 0.05) than that in stage 1.

**Table 1:** Isolation of *S. aureus* from “animal, environment, and human” samples in the broiler feeding cycle.

		Stage 1			Stage 2			
Source	Sample type	Number of samples	Positive detection	Isolation rate (%)	Number of samples	Positive detection	Isolation rate (%)	*p*-value
Animal	Chicks	150	0	0	0	0	0	/
	Broilers	0	0	0	270	83	30.74	/
		150	0	0	270	83	30.74	0.000^**^
Environment	Air	30	0	0	40	10	25.00	0.003^**^
	Water	15	0	0	45	10	22.22	0.046^*^
	Trough/cage	120	1	0.83%	170	72	42.35	0.000^**^
	Floor	60	0	0	60	20	33.33	0.000^**^
	Fly	27	0	0	49	0	0	/
	Transport	0	0	0	25	2	8.00	/
	Feed	5	0	0	25	1	4.00	0.649^ns^
	Feces	15	0	0	30	1	3.33	0.475^ns^
	Soil	15	1	6.67%	20	1	5.00	0.833^ns^
		287	2	0.69%	464	117	25.21	0.000^**^
Human	Throat swabs	8	2	25.00%	19	12	63.16	0.070^ns^
	Hand swabs	8	2	25.00%	19	9	47.37	0.280^ns^
		16	4	25.00%	38	21	55.27	0.042^*^
Total		453	6	1.32%	772	221	28.63	0.000^**^

^*^P < 0.05; ^**^P < 0.01.

### 4.2 Antimicrobial sensitivity profile of *S. aureus* in the broiler feeding cycle

**Figure 1:**
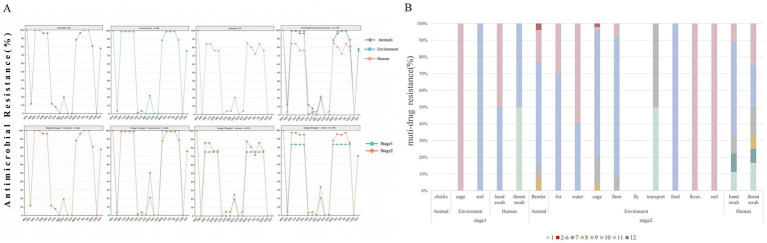
Antimicrobial resistance pattern of *S. aureus* from animal, environment, and human sources in the broiler feeding cycle. **(A)** Row 1 shows the overall resistance results of *S. aureus* from the three sources. Row 2 shows the comparison of the resistance results of strains from the three sources during stage 1 and stage 2. The antibiotics in each row from left to right are penicillin (PEN), amoxicillin/clavulanic acid (AMC), erythromycin (ERY), clindamycin (CLI), enrofloxacin (ENR), ofloxacin (OFL), ceftiofur (CEF), cefoxitin (CFX), sulfafurazole (SIS), oxacillin(OXA), vancomycin(VAN), sulfamethoxazole (SXT), doxycycline (DOX), florfenicol (FFC), tiamulin (TIA), tilmicosin (TIL), gentamicin (GEN), linazolamide (LZD), and tetracycline (TET). **(B)** Distribution of multidrug resistance patterns among 139 isolates. The different colored blocks represent the extent of multidrug resistance.

The analysis of the antimicrobial resistance spectrum of 139 representative strains revealed that the multidrug resistance rate was as high as 96.4% (134 out of 139). Drug resistance was mainly distributed among 9 to 11 different antibiotics (Figure 1B). Notably, the resistance rates to 9 drugs were over 80%, including erythromycin, clindamycin, ceftiofur, fosfomycin, tylosin, doxycycline, enrofloxacin, ofloxacin, and tamoxifen (Figure 1A; Table 2). On the contrary, it was observed that the drug resistance rates to amoxicillin/clavulanic acid, cefotaxime, cefoxitin, sulfonamide, and cotrimoxazole were less than 10%. Furthermore, vancomycin or linezolid-resistant strains were not isolated (Table 2).

**Table 2:** Drug resistance of *S. aureus* in “animal, environment, and human” sources in the broiler feeding cycle.

Drugs	Animal (*n* = 26)		Environment (*n* = 88)					Human (*n* = 25)					Total
	Stage2 (26)		Stage1 (2)		Stage2 (86)		*P*-value	Stage1 (4)		Stage2 (21)		*P*-value	*P*-value
PEN	26	100%	2	100%	86	100%	/	4	100%	21	100%	/	/
AMC	3	11.54%	0	0	3	3.49%	0.788^ns^	0	0	0	0	/	0.101^ns^
ERY	26	100%	2	100%	85	98.84%	0.878^ns^	3	75%	18	85.71%	0.592^ns^	0.001^**^
CLI	26	100%	2	100%	85	98.84%	0.878^ns^	3	75%	18	85.71%	0.592^ns^	0.001^**^
ENR	25	96.15%	2	100%	85	98.84%	0.878^ns^	3	75%	16	76.19%	0.959^ns^	0.000^**^
OFL	25	96.15%	2	100%	85	98.84%	0.878^ns^	3	75%	16	76.19%	0.959^ns^	0.000^**^
CEF	3	11.54%	0	0	1	1.16%	0.878^ns^	0	0	0	0	/	0.013^*^
CFX	2	7.69%	0	0	3	3.49%	0.788^ns^	0	0	1	4.76%	0.656^ns^	0.638^ns^
SIS	0	0	0	0	0	0	/	0	0	1	4.76%	0.656^ns^	0.101^ns^
OXA	5	19.23%	1	50.00%	18	20.93%	0.323^ns^	1	25%	4	19.05%	0.785^ns^	0.960^ns^
VAN	0	0	0	0	0	0	/	0	0	0	0	/	/
SXT	0	0	0	0	0	0	/	0	0	1	4.76%	0.656^ns^	0.101^ns^
DOX	15	88.24%	2	100%	37	88.10%	0.604^ns^	3	75%	14	87.50%	0.531^ns^	0.91^ns^
FFC	25	96.15%	2	100%	85	98.84%	0.878^ns^	3	75%	17	80.95%	0.785^ns^	0.001^**^
TIA	26	100%	2	100%	85	98.84%	0.878^ns^	3	75%	15	71.43%	0.884^ns^	0.000^**^
TIL	26	100%	2	100%	85	98.84%	0.878^ns^	3	75%	18	85.71%	0.592^ns^	0.001^**^
GEN	21	80.77%	2	100%	76	88.37%	0.608^ns^	3	75%	16	76.19%	0.959^ns^	0.241^ns^
LZD	0	0	0	0	0	0	/	0	0	0	0	/	/
TET	7	77.78%	/	/	34	77.27%	/	0	/	0	0	/	/

Doxycycline: 17 broiler, 44 environment, and 20 human strains were tested; tetracycline: 9 broiler, 44 environment, and 5 human strains were tested. *p < 0.05; **p < 0.01.

Among the various feeding stages, the multidrug resistance rate in stage 1 was 83.33% (5/6), and 10–11 drug resistance categories were mainly observed. In contrast, the stage 2 strains showed a significantly higher multidrug resistance rate of 97% (129 out of 133), with resistance primarily concentrated in the 9–11 drug resistance category (Figure 1B). The resistance rate of *S. aureus* to eight antibiotics increased during stage 2, including erythromycin, clindamycin, enrofloxacin, ofloxacin, doxycycline, fosfomycin, tylosin, and tamoxifen. However, the difference was not statistically significant (*p* > 0.05) (Figure 1A; Table 2).

Among the three sources, the multidrug resistance rate of broilers was 100% (26/26), that of environmental sources was 98.86% (87/88), and that of human sources was 84% (21/25). The multidrug resistance rates of animal and environmental sources were mainly distributed across 8–11 levels, while that of human sources was mainly distributed in 1 and 7–11 levels (Figure 1B). The resistance rates of broiler and environmental strains to drugs including erythromycin, clindamycin, enrofloxacin, ofloxacin, florfenicol, tylosin, and tamoxifen were over 90%, which was significantly higher than that observed in human strains (*p* < 0.01). Additionally, the resistance rates of broiler strains to amoxicillin/clavulanic acid and ceftiofur were approximately 11%, while that of environmental strains was 1%, and human strains were not resistant to these drugs. Notably, the resistance to sulfisoxazole and cotrimoxazole was observed exclusively in human strains (Figure 1A; Table 2).

### 4.3 MLST of *S. aureus* in the broiler feeding cycle

**Figure 2:**
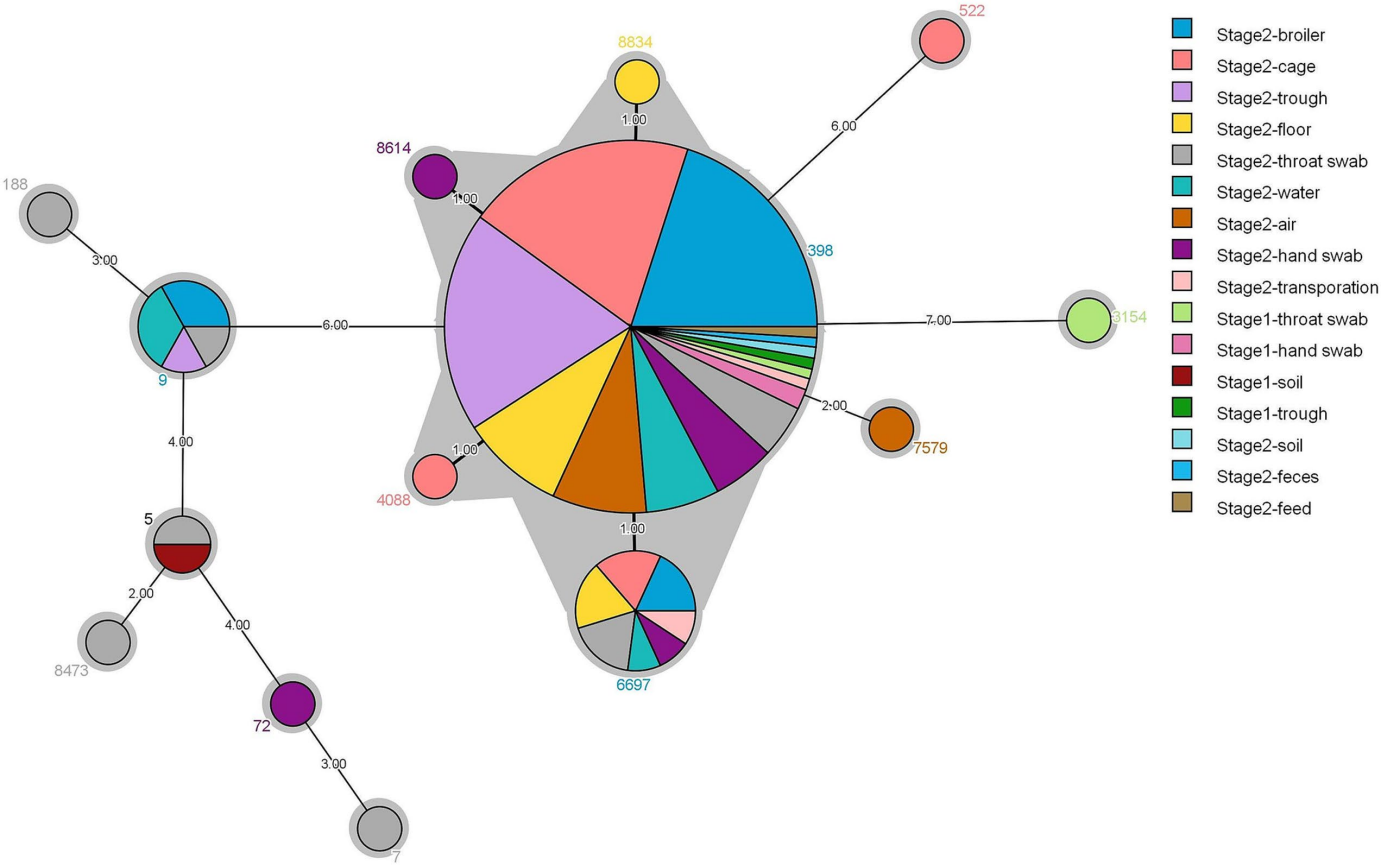
Minimum spanning tree for 139 strains of *S. aureus* based on different types of samples and multilocus sequence typing.

Of 139 representative strains, 14 sequence types (STs) were identified, with the dominant types being ST398 (79.13%), ST6697 (7.91%), and ST9 (4.31%) (Figure 2). Stage 1 contained three STs, ST398 (66.66%), ST5 (33.2%), and ST3154 (33.2%). Stage 2 contained 11 STs, with the main types being ST398 (79.69%), ST6697 (8.27%), and ST9 (4.51%). ST398 was the most widely distributed strain, found in 11 different sample types, including broiler and environmental sources (air, feed, water, troughs/cages, floors, transport, feces, and soil), as well as human sources (throat swabs and hand swabs). This was followed by ST6697, which differed from ST398 by only one allele and was obtained from seven sample types: broiler, environmental (ground, cage, water, and transport), and human (throat swabs and hand swabs) sources. Additionally, ST8834 (ground), ST8614 (hand swabs), and ST40883 (troughs/cages) were each only one allele away from ST398 and were exclusively found in a single sample type. ST9 MRSA strains were derived from four types: broiler, environmental (troughs and drinking water), and human (throat swabs) sources.

### 4.4 SNP evolutionary analysis of *S. aureus* in the broiler feeding cycle

**Figure 3:**
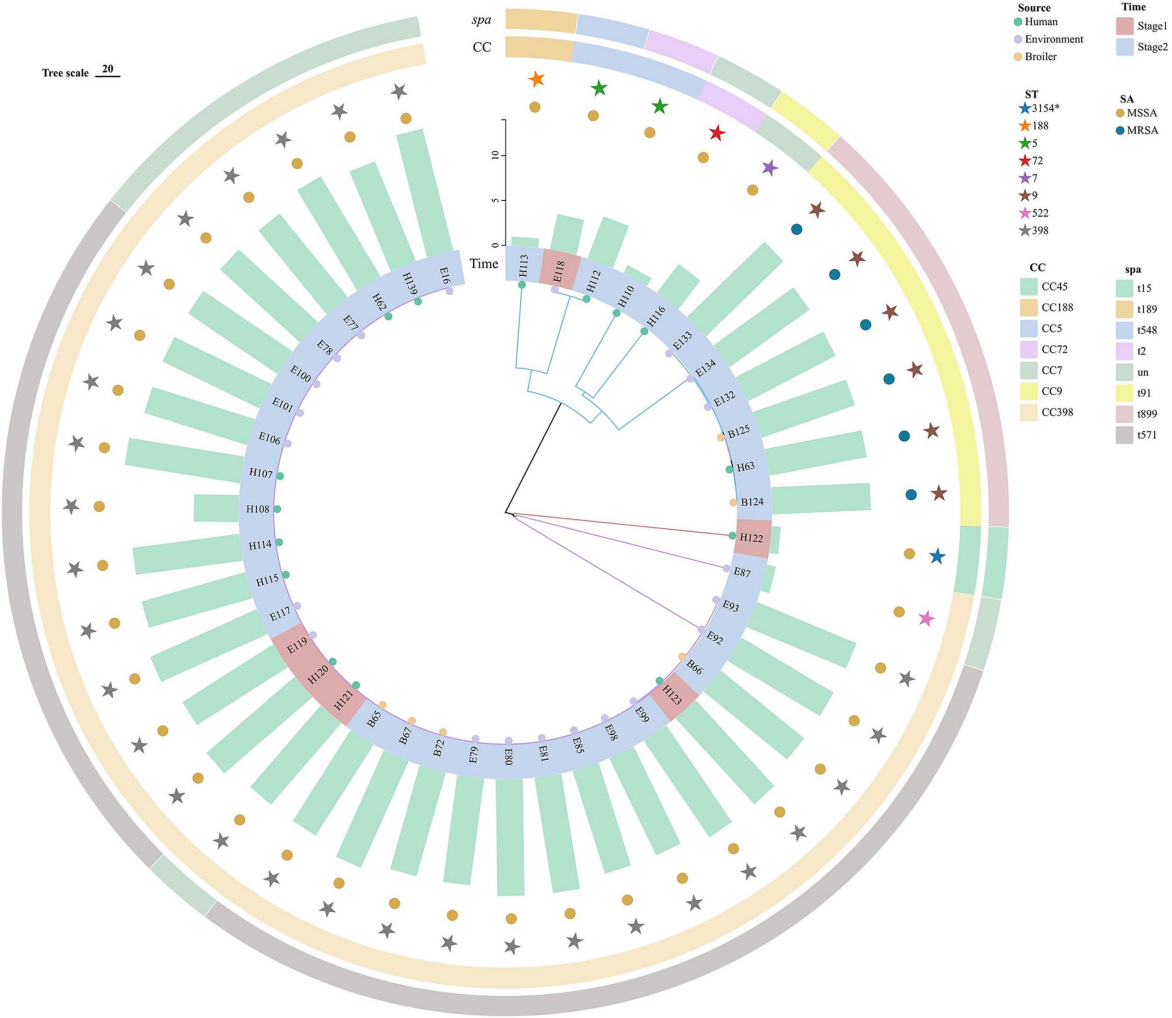
Evolutionary tree of the core genome of *S. aureus* (*N* = 42) in “animal, environment, and human” sources in the broiler feeding cycle. From inside to outside, the first circle represents the animal, environment, and human sources, the second circle represents strains from stage 1 and stage 2, the third circle represents the number of strains carrying resistance genes, the fourth circle represents MSSA and MRSA, the fifth circle represents different ST types, the sixth circle represents different clonal groups, and the outermost circle represents different *spa* types.

The SNP evolution tree was constructed for 42 representative strains. All strains were divided into three branches with the number of SNPs ranging from 1 to 37,761 (Figure 3). Clade I (red) was the closest to the root node, with only one human strain from stage 1, which differed by at least 37,761 SNPs from other strains. Clade II (blue) contained 10 strains from stage 2 and 1 strain from stage 1. Six MRSA strains were clustered in the same sub-clade, all of which were ST9-t899 type. The loci differences between B124 and B125 with E134 were 34 and 52, respectively. Compared to the other strains in Clade II, all six MRSA strains carried a large number of resistance genes. The two strains from stage 1 (soil) and stage 2 (throat swabs), although clustered in the same sub-branch and both ST5, exhibited greater differences in loci and possessed fewer resistance genes. Clade III (purple) was the furthest from the root node, containing stage 1 (4/30,13.33%) and stage 2 (26/30,86.67%) strains, all of which belonged to the CC398 clonal complex (ST398, ST522) and were clustered in the same sub-clade. The strains from both stages differed in loci ranging from 1 to 541. H120 (stage 1) differed from B67 and E99 (stage 2) by 9 loci and from H107 by only 1 SNP. E119 (stage1) differed from E100 and B72 (stage 2) by SNP distances of 15 and 8, respectively. In addition, several strains with SNP distances of less than 10 were found between the three sources, including B67 and E81, B67 and H107, H107 and H120, H120 and E80, E98 and E99.

### 4.5 Drug-resistant gene profiles of *S. aureus* in the broiler feeding cycle

**Figure 4:**
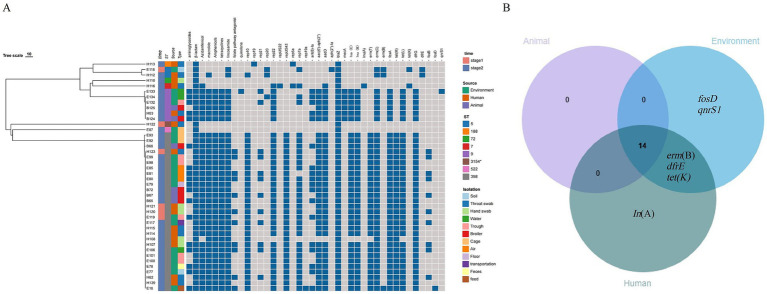
**(A)** Analysis of antibiotic-resistance phenotypes of *S. aureus* (*n* = 42) and the drug-resistance genes and plasmid replicons it carries during the broiler feeding cycle. **(B)** Venn diagram of drug-resistance genes of *S. aureus* strains from different sources.

A total of 20 antibiotic resistance genes were identified in 42 representative strains, including tetracyclines (*n* = 3), aminoglycosides (*n* = 3), macrolides (*n* = 3), lincosamides (*n* = 3), folate pathway antagonists (n = 2), *β*-lactams (*n* = 2), fluorfenicol (*n* = 1), fosfomycin (*n* = 2), and quinolones (*n* = 1) (Figure 4A). Notably, 100% of the strains possessed the *β*-lactam gene *blaZ*, and over 80% of the strains exhibited nine resistance genes, namely *ant(6)-Ia*, *aac(6′)-aph(2″)*, *aadD*, *lsa*(E), *lnu*(B), *erm*(C), *fexA*, *tet*(L), and *dfrG*.

#### 4.5.1 Distribution of drug resistance genes at different feeding stages

The strains in stage 1 and stage 2 carried 16 and 20 drug resistance genes, respectively, and there was no statistically significant difference in the average number of antibiotic resistance genes (*p* > 0.05). Broiler, environmental, and human strains carried 14, 19, and 18 drug resistance genes, respectively, and the mean values of the number of resistance genes carried by the strains were 11.84, 11.28, and 8.53, respectively, with no statistically significant difference (*p* > 0.05). However, the number of resistance genes carried by environmental strains (11.63) in stage 2 was significantly higher than that in the human source (8.63), but there was no significant difference between the strains from the broiler and the other two sources (*p* > 0.05).

#### 4.5.2 Distribution of drug resistance genes in different sources

The three source strains collectively contained 14 resistance genes. Some of these genes are specific to certain strains. *Lnu*(A) was the unique resistance gene found in the human source strain, while *erm*(B) and *tet*(K) were unique to both the environmental and human source strains. Additionally, the 14 resistance genes carried by the broiler were also observed in both the environmental and human sources (Figure 4B). Furthermore, certain resistance genes were restricted to specific sequence types (STs). For instance, the β-amide resistance gene *mecA* was exclusively found in ST9 strains (6 out of 6), the phosphomycin resistance gene *fosB* was limited to ST398 strains (10 out of 29), and the amido-alcohol resistance gene *dfrE* was solely detected in ST5 strains (2 out of 2).

### 4.6 Mobile genetic elements of *S. aureus* in the broiler feeding cycle

Ten plasmid replicons were detected in the 42 representative strains, among which the most widely distributed plasmid replicon belongs to the type rep22 (35 out of 42) plasmid, followed by the types rep10 (30 out of 42), repUS43 plasmid (29 out of 42), and rep21 (11 out of 42). Furthermore, three transposon elements, Tn*558* (35 out of 42), Tn*6009* (29 out of 42), and Tn*551* (2 out of 42), were detected in all strains. There were seven and nine plasmid replicon types in environmental and human strains, respectively, which had more genetic diversity than broiler strains (four types). Notably, no transposable elements associated with resistance transmission were identified in one environmental strain (cage) and two human strains (throat and hand swabs).

**Figure 5:**
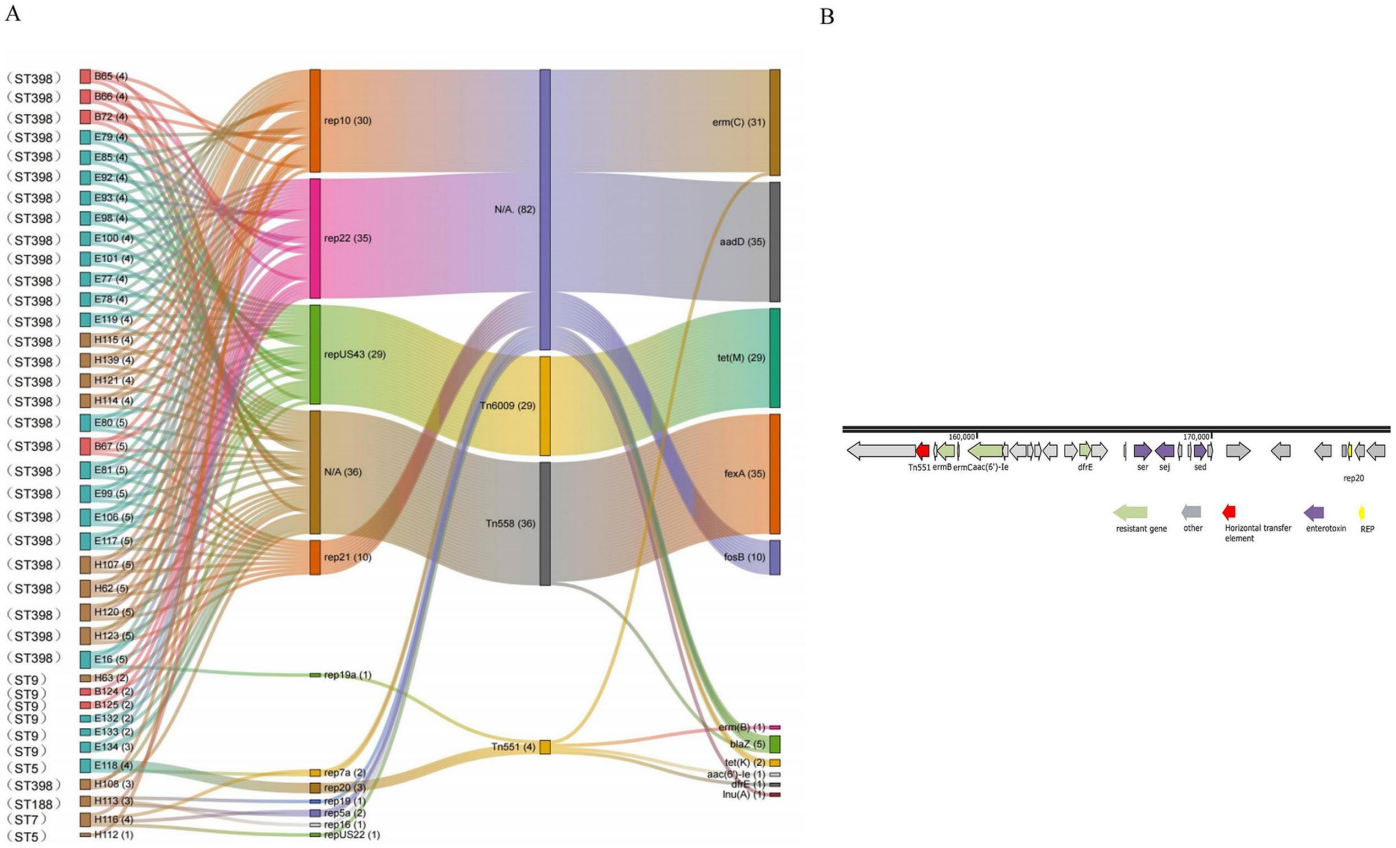
**(A)** Sankey map of *S. aureus* (*N* = 42) carrying mobile genetic elements and its resistance genes in the broiler feeding cycle (E87, E110, and H122 do not contain removable elements and are therefore not shown in the diagrams). **(B)** Genetic background map of the E118 strain.

The plasmid replicons carried by different strains were obviously associated with various genetic determinants. The same ST strain carries multiple mobile genetic elements, with all ST398 strains containing three plasmids (rep22, rep10, and repUS43) and two transposons (Tn*6009* and Tn*558*). The plasmids rep22 and rep10 harbored the resistance genes *aadD* and *erm*(C), while repUS43 carries the Tn*6009* transposon linked to *tet*(M), and Tn*558* transposon is associated with the *fexA* resistance gene. The ST9 MRSA includes two strains from the broiler, three strains from the environment, and one additional human strain, all of which had one plasmid (rep22) and one transposon (Tn*558*) linked to the resistance genes *aadD* and *fexA* (Figure 5A).

Some plasmids were identified in specific ST strains. The rep21 plasmid (feed) and the rep19a plasmid (broiler, environment, and human) were found in certain ST398 strains and were associated with the *erm*(B) and *fosB* resistance genes. The rep20 plasmid and the Tn*551* transposon were detected in the ST5 strain (soil), where Tn*551* was coupled to the *erm(C)*, *erm(B)*, and *aac(6′)-Ie* resistance gene clusters (Figure 5B). Additionally, the rep16, rep19, and rep5a plasmids were simultaneously identified in the strain ST188 (throat swabs), and all these plasmids carried the *blaZ* resistance gene. Furthermore, the repUS22 plasmid, which carries the *lnu*(A) resistance gene, was found exclusively in strain ST7 (throat swabs) (Figure 5).

## 5 Discussion

Foodborne pathogens and drug resistance have become major problems affecting livestock breeding, food safety, and public health, and have posed considerable challenges for veterinary and human medicine, as well as the environment. Since 2018, the FAO, WHO, and WOAH have been working together to address the risks of AMR to humans, animals, and the environment through the concept of “One Health.” In this study, we investigated and analyzed the drug resistance and transmission factors of the foodborne pathogen *S. aureus* through the animal–environment–human chain during the feeding cycle of broilers. It was the first time to investigate and analyze the factors influencing the spread of *S. aureus* and the evolution of drug resistance in broiler farming based on the concept of “One Health.”

The results of *S. aureus* isolation showed that the target bacteria were not isolated from 1-day-old chick samples, which indicated that the bacteria may not have colonized the introduced chicks. In stage 1, the isolation rate of *S. aureus* was 1.32%, of which two strains were from environmental samples (trough/cage and soil) and four strains were from human samples (hand swabs and throat swabs), indicating that the farm strictly implemented measures such as hygiene management and disinfection, resulting in a low isolation rate of target bacteria at stage 1. However, after a feeding cycle, due to inadequate biosafety measures, the target organisms multiplied rapidly. In this study, the isolation rate of *S. aureus* (30.74%) from broiler was significantly higher than that reported in Chongqing, China (6.7%) (Dong et al., 2023), yet lower than that observed in Indonesia (38.52%) (Hermana et al., 2023). The variation in isolation rates can be attributed to differences in sample types—throat swab samples for the former and cloacal swab samples for the latter—or to differences in feeding management practices. These findings further confirm the existence of epidemiological differences across various regions.

Based on the MLST and SNP evolution analyses, ST398 and ST5 were the cross-typing of the strains in stage 1 and stage 2, and ST398, ST6697, and ST9 were the cross-typing of the animal, environment, and human sources. All the ST398 strains were clustered in the same sub-branch. The SNP loci difference between the stage 1 strain of the human source (H120) and the stage 2 strains of the animal (B67), environmental (E99), and human sources (H107) were less than 10; the stage 1 environmental source strain (E119) differed from the stage 2 environmental source (E100) and broiler source strain (B72) by 15 and 8 difference sites, respectively, which indicated that the strains of different feeding stages and sources were highly related to each other and confirmed that the environmental remnants of the bacteria and the worker carriage may be the influential factors leading to the colonization and transmission of the foodborne pathogen *S. aureus* during broiler feeding, especially worker carriage, with the highest isolation rates among all types of samples for both stages of human samples (25.00 and 55.27%). A study conducted in Bangladesh reported that 65% of farm workers carried *S. aureus* (Hossain et al., 2022), which is higher than the current results. A Norwegian study found that workers who were in close contact with livestock on farms can transmit *S. aureus* to other farms (Elstrøm et al., 2019). The present study found that worker carriage could be transmitted to the next stage of farming, suggesting that worker carriage is a key risk factor for the spread of pathogens. It is worth noting that six ST9 MRSA strains were also isolated during stage 2 in this study. Zhang Wei (Zhang et al., 2024) found the chicken source ST9-t899 MRSA in Hanzhong, and Zhang Tengfei also found ST9-t899 MRSA contained in chicken farms in Wuhan (Zhang et al., 2021), suggesting that ST9-t899 MRSA strains are prevalent and spread among broilers in different regions of China. The six ST9 MRSA strains in this study were from broiler, environmental (drinking water and trough), and human (throat swabs) source samples and clustered in the same sub-branch of the SNP evolutionary tree, in which the difference site between the animal source strain (B124) and the environmental source strain (E134) was only 34, which is a relatively close affinity. This further confirms that MRSA strains can be transmitted between animal, environmental, and human sources, but how they are transmitted needs to be further investigated and researched in the future.

The results of the drug sensitivity test showed that the multidrug resistance rate of *S. aureus* in stage 1 was 83.33%, and the resistance rates to nine drugs were more than 80%, including erythromycin, clindamycin, enrofloxacin, ofloxacin, doxycycline, flucytosine, tylosin, tamoxifen, and gentamicin, which indicated that the *S. aureus* isolated during stage 1 was insensitive to antibiotics. In stage 2, the multidrug resistance rate of *S. aureus* was 97%, with increased resistance to eight drugs: erythromycin, clindamycin, enrofloxacin, ofloxacin, doxycycline, florfenicol, tylosin, and tilmicosin; the multidrug resistance rates of the strains of the broiler (100%) and environmental sources (98.86%) was significantly higher than that of the human source (84.0%) (*P* < 0.01), which may be due to the selection pressure of the use of antibiotics in the feeding process. These results further confirmed that not only is *S. aureus* circulating in the animal, environment, and human sources after a feeding cycle but antibiotic resistance is also being transmitted. The whole gene-sequencing analysis of 42 representative strains showed that there was no significant difference in the average number of resistance genes carried at stage 1 and stage 2 (*P* > 0.05). Notably, 100% of broiler source strains, 90% of environmental source strains, and 60% of human source strains carried more than 10 resistance genes, which was also consistent with the results of drug sensitivity experiments. All strains contained a total of 19 resistance genes: 19 from environmental sources, 18 from human sources, and 14 from broilers. Notably, the resistance genes from broilers were also present in both environmental and human sources, suggesting a potential for cross-transmission and frequent exchange of resistance genes between environmental residues and workers, which may subsequently lead to transmission to animals through close contact. The present study also found that 26.19% (10/42) of the ST398-type strains carried the resistance to fosfomycin, a prohibited drug for animals, which was comparable to the 30.1% positive rate of the *fosB* resistance gene among broiler-derived *S. aureus* in Guangdong (Hu et al., 2023), in which the antibiotic resistance genes were found only in H120 and H123 during stage 2, and the antibiotic resistance genes were found in the broiler (B67) and environmental strains (H99, H98) with less than 10 loci differences, suggesting that the isolates carrying the resistance gene *fosB* may have been transmitted by workers during the broiler feeding cycle. Bacteria isolated from farm workers in close contact with food animals have been found to be highly resistant in several studies (Nadimpalli et al., 2016; Peng et al., 2022). Additionally, many studies have reported that humans can act as reservoirs for *S. aureus*, introducing it into the farm environment. Therefore, the issue of worker carriage should be considered critical (Goerge et al., 2017; Schulz et al., 2019).

Plasmid replicons and transposons are significant mobile genetic elements. Mobile element analyses showed that environmental and human strains carry more types of plasmid replicons that mediate drug resistance than animal sources and are genetically diverse; ST398 and ST9 bacteria carry multiple identical plasmids and transposons. Notably, all ST398-type strains contained three plasmids—rep22, rep10, and repUS43—as well as the transposon Tn*558*, which carried the *aadD*, *erm*(C), *tet*(M), and *fexA* antibiotic resistance genes, respectively. Furthermore, the environmental and human-derived strains in stage 1 exhibited a strong relationship with those in stage 2, indicating that these resistance genes may have been transferred to the subsequent stage through human and environmental source strains and transmitted horizontally between the three source strains via mobile elements. Similarly, the ST9 strains possessed both the rep22 plasmid and Tn*558*, which carried the *aadD* and *tet*(M) resistance genes, showing minor loci differences between the animal (B124) and environmental source strains (E134). This revealed the horizontal transmission of the potential resistance gene among the strains. An investigation into on-farm MRSA of porcine and human origin demonstrated that the rep22 and repUS43 plasmids carry the *aadD* and *tet*(M) resistance genes (Narongpun et al., 2023), which aligns with the findings of this study.

In summary, this study investigated and analyzed the foodborne pathogen *S. aureus* and its drug resistance throughout the broiler feeding cycle by MLST and WGS technology. The findings indicate that multidrug-resistant *S. aureus*, persisting in the environment and being carried by farm workers during stage 1, may contribute to the dissemination of target *S. aureus* during the feeding cycle. This is particularly relevant for practitioners, who play a crucial role in the colonization and spread of these pathogens. These results highlight the deficiencies in current biosecurity measures and emphasize the importance of adopting a holistic approach to addressing foodborne pathogens and their resistance. It is imperative to establish the concept of “One Health” and identify key influencing factors to prevent the further spread of foodborne pathogens and their resistance.

## Data Availability

The whole gene sequencing results of all strains were submitted to GenBank 16 database and obtained BioProject ID PRJNA1184813.

## Footnotes

^1^ http://www.mlst.net

^2^ https://cge.cbs.dtu.dk/services/

^3^ https://cge.food.dtu.dk/services/spaTyper/

^4^ https://www.chiplot.online/

^5^ http://bacdb.cn/BacWGSTdb/index.php

^6^ https://www.chiplot.online/normalTree.html
